# A rare cause of atraumatic fractures: case series of four patients with tumor-induced osteomalacia

**DOI:** 10.1186/s40842-020-00101-8

**Published:** 2020-07-06

**Authors:** Debbie W. Chen, Gregory A. Clines, Michael T. Collins, Liselle Douyon, Palak U. Choksi

**Affiliations:** 1 Division of Metabolism, Endocrinology and Diabetes, University of Michigan, Ann Arbor, MI USA; 2 Ann Arbor Veterans Affairs Healthcare System, Ann Arbor, MI USA; 3 Skeletal Disorders and Mineral Homeostasis Section, National Institute of Dental and Craniofacial Research, NIH, Bethesda, MD USA

**Keywords:** Osteomalacia, Fractures, Hypophosphatemia, Fibroblast growth factor 23

## Abstract

**Background:**

Tumor-induced osteomalacia (TIO) is a rare paraneoplastic syndrome that presents with hypophosphatemia, bone pain, muscle weakness and fractures. We report a case series of four patients with TIO that resulted in significant muscle weakness and multiple atraumatic fractures.

**Case presentation:**

Four patients were referred to an endocrinology clinic for the evaluation of multiple atraumatic fractures, muscle weakness, generalized muscle and joint pain. Laboratory evaluation was notable for persistent hypophosphatemia due to urinary phosphate wasting, low to low-normal 1,25-dihydroxyvitamin D, elevated alkaline phosphatase and elevated fibroblast growth factor 23 (FGF23). Tumor localization was successful, and all four patients underwent resection of phosphaturic mesenchymal tumors. Post-operatively, patients exhibited normalization of serum phosphorus, in addition to significant improvement in their ambulatory function.

**Conclusion:**

Hypophosphatemia with elevated FGF23 and low 1,25-dihydroxyvitamin D level in the setting of multiple atraumatic fractures necessitates careful evaluation for biochemical evidence of tumor-induced osteomalacia.

## Background

Tumor-induced osteomalacia (TIO), also known as oncogenic osteomalacia, is a rare paraneoplastic syndrome that presents with bone pain, muscle weakness, and fractures [1]. Hypophosphatemia, phosphaturia and elevated fibroblast growth factor 23 (FGF23) are hallmark laboratory abnormalities of this syndrome [2, 3]. Hypophosphatemia and inappropriately low to normal 1,25-dihydroxyvitamin D level in patients with atraumatic fractures is concerning for TIO [4]. We report a case series of four patients from a single institution who were referred for unexplained fractures and subsequently diagnosed with TIO.

## Case presentation

### Case 1

**Fig. 1:**
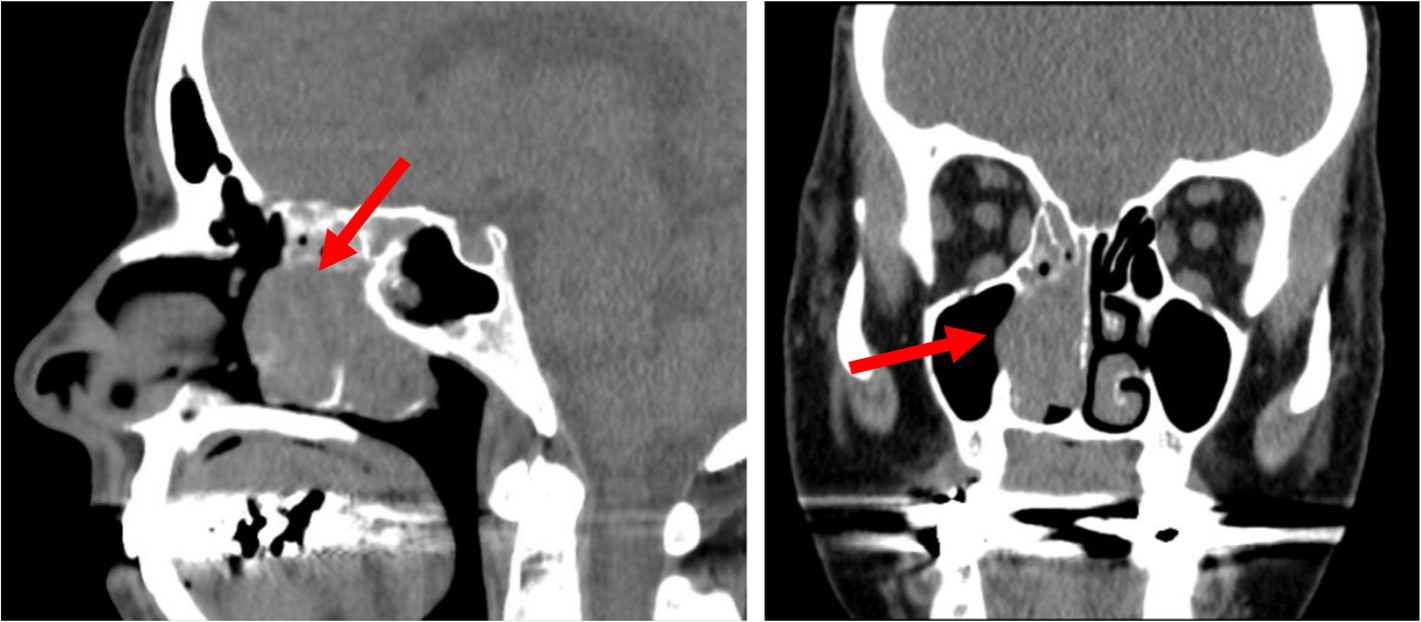
Maxillofacial CT scan of patient in case 1 demonstrated a large polypoid mass in the right middle turbinate (red arrows)

A 59-year-old postmenopausal female with a history of hypothyroidism initially presented to her primary care physician for diffuse bone pain, proximal muscle weakness manifested as difficulty standing up from a seated position, and pain when walking distances of less than one mile. Laboratory evaluation was notable for elevated intact parathyroid hormone (iPTH, 143 pg/mL; normal range 10 to 65 pg/mL), normal serum calcium (10.0 mg/dL; normal range 8.6 to 10.3 mg/dL), hypophosphatemia (1.8 mg/dL; normal range 2.7 to 4.6 mg/dL), elevated alkaline phosphatase (165 IU/L; normal range 40 to 116 IU/L), and normal thyroid stimulating hormone (2.70 mIU/L; normal range 0.30 to 5.50 mIU/L). She was diagnosed with normocalcemic primary hyperparathyroidism and underwent a subtotal parathyroidectomy with normalization of the iPTH level. Pathology was consistent with hypercellular left superior and inferior glands. Five months postoperatively, when calcitriol therapy was discontinued, she continued to have left hip pain and required a cane for ambulation. Due to recurrently elevated iPTH level (108 pg/mL) and new low-trauma bilateral subtrochanteric hip fractures, she was started on bisphosphonate therapy and referred to the University of Michigan Endocrinology clinic. Eight months postoperatively, at her initial endocrinology visit, repeat biochemical evaluation was remarkable for normal iPTH level, persistent hypophosphatemia, low-normal 1,25-dihydroxyvitamin D, elevated alkaline phosphatase, and elevated plasma FGF23 (Table 1). Calculated tubular maximum for phosphate corrected for glomerular filtration rate (TmP/GFR) of 1.31 mg/dL (normal range 2.5 to 4.2 mg/dL) confirmed renal phosphate wasting (Table 2). She was prescribed phosphorus supplementation, restarted on calcitriol therapy and advised to discontinue bisphosphonate therapy. A ^18^F-fluorodeoxyglucose positron emission tomography/computed tomography (^18^F-FDG PET/CT) scan was unrevealing. Maxillofacial CT scan demonstrated a large polypoid mass in the right middle turbinate (Fig. 1). Biopsy of the right nasal mass revealed a low-grade spindle cell neoplasm, consistent with a phosphaturic mesenchymal tumor. She subsequently underwent endoscopic excision of the right nasal tumor. One month postoperatively, phosphorus and calcitriol supplementation were discontinued with subsequent normalization of the serum phosphorus (4.4 mg/dL) and FGF23 (94 RU/mL) levels. She had no additional fractures, and her functional status significantly improved such that she was able to ambulate pain-free without assistive devices.

**Table 1:** Laboratory evaluation of patients at the time of evaluation was notable for hypophosphatemia, low to low-normal 1,25-dihydroxyvitamin D level, and elevated fibroblast growth factor 23 (FGF23) level. Abnormal laboratory values are annotated with (H) if above the normal range and (L) if below the normal range

	Case 1	Case 2	Case 3	Case 4
**Serum phosphorus** (2.7–4.6 mg/dL)	1.7 (L)	1.0–1.5 (L)	1.3–1.5 (L)	2.1 (L)
**Serum creatinine** (0.5–1.0 mg/dL)	0.85	0.85	0.87	0.97
**Serum calcium** (8.6–10.3 mg/dL)	10.0	9.4	9.9	9.0
**Serum albumin** (3.5–4.9 g/dL)	4.6	4.5	4.3	4.0
**Intact parathyroid hormone** (10–65 pg/mL)	25	61	113 (H)	46
**25-hydroxyvitamin D** (25–100 ng/mL)	52	18 (L)	24 (L)	27
**1,25-dihydroxyvitamin D** (18–78 pg/mL)	18	7 (L)	13 (L)	20
**Alkaline phosphatase** (40–116 IU/L)	145 (H)	262–278 (H)	246–326 (H)	441 (H)
**Plasma FGF23** (< 180 RU/mL)	264 (H)	540 (H)	2189 (H)	548 (H)

**Table 2:** Evidence of renal phosphate wasting as determined by calculation of tubular maximum for phosphate corrected for glomerular filtration rate (TmP/GFR). The patient in case 3 had a 24-h urine phosphorus measurement of 900 mg/24 h, but urine creatinine measurement was not obtained, so TmP/GFR was not calculated

	Case 1	Case 2	Case 4
**Serum phosphorus** (2.7–4.6 mg/dL)	1.7	1.3	1.8
**Serum creatinine** (0.5–1.0 mg/dL)	0.77	0.85	0.90
**Urine phosphorus** (mg/dL)	24.1		35.1
**Urine phosphorus** (400–1200 mg/24 h)	653.1	990.0	446.8
**Urine creatinine** (mg/dL)	46.5		86.1
**Urine creatinine** (1.0–1.8 g/24 h)	1.3	1.7	1.1
**Calculated TmP/GFR** (2.5–4.2 mg/dL)	1.3	0.8	1.4

### Case 2

**Fig. 2:**
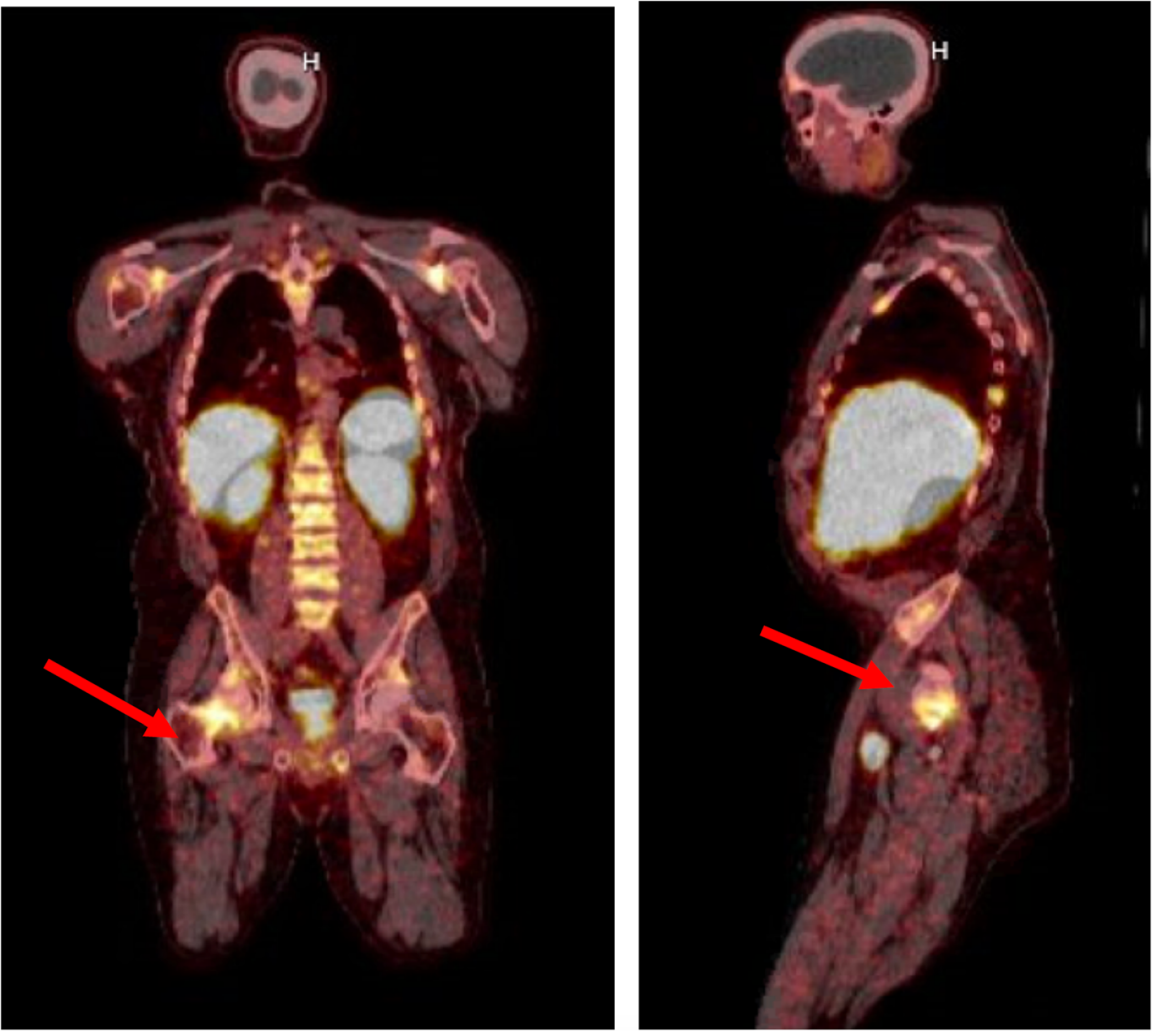
^68^Ga-DOTATATE PET/CT scan of patient in case 2 demonstrated marked tracer uptake in a 1.9 × 1.3 cm soft tissue mass underlying the right sartorius muscle (red arrows)

A 44-year-old male veteran with no significant medical history presented with a two-year history of persistent left knee pain despite treatment with intra-articular glucocorticoids and meniscus repair. Laboratory evaluation was remarkable for hypophosphatemia and elevated alkaline phosphatase (Table 1). Magnetic resonance imaging (MRI) revealed multiple new tibial stress fractures. Dual-energy x-ray absorptiometry demonstrated evidence of low bone mass, prompting treatment with denosumab. Due to worsening knee pain, denosumab was discontinued after the first dose and he was transitioned to conservative therapy with vitamin D and calcium supplementation. Over the subsequent year, he developed progressive generalized diffuse joint pains in the upper extremities bilaterally and muscle weakness requiring crutches for ambulation. He was eventually evaluated by an oncologic orthopedic surgeon for these multiple fractures. He was noted to have persistent hypophosphatemia, low 1,25-dihydroxyvitamin D, elevated alkaline phosphatase, and elevated plasma FGF23 (Table 1). Calculated TmP/GFR of 0.80 mg/dL confirmed renal phosphate wasting (Table 2). Due to concern for TIO, he was referred to the University of Michigan Endocrinology clinic for further evaluation. PET scan showed an enlarged left level III lymph node in the neck, but this was reported benign by pathologic examination after biopsy. He was initiated on phosphorus and calcitriol supplementation with weekly monitoring of serum calcium and phosphorus levels. A ^68^Ga-DOTATATE PET/CT scan performed for tumor localization revealed marked tracer uptake in a 1.9 × 1.3 cm soft tissue mass underlying the right sartorius muscle (Fig. 2). He underwent surgical excision of this soft tissue mass and pathology confirmed it as a phosphaturic mesenchymal tumor. Three weeks post-operatively, his functional status improved, and he was able to ambulate with a walker. Six weeks post-operatively, phosphorus and calcitriol supplementation were discontinued with subsequent normalization of the serum phosphorus level (4.1 mg/dL).

### Case 3

**Fig. 3:**
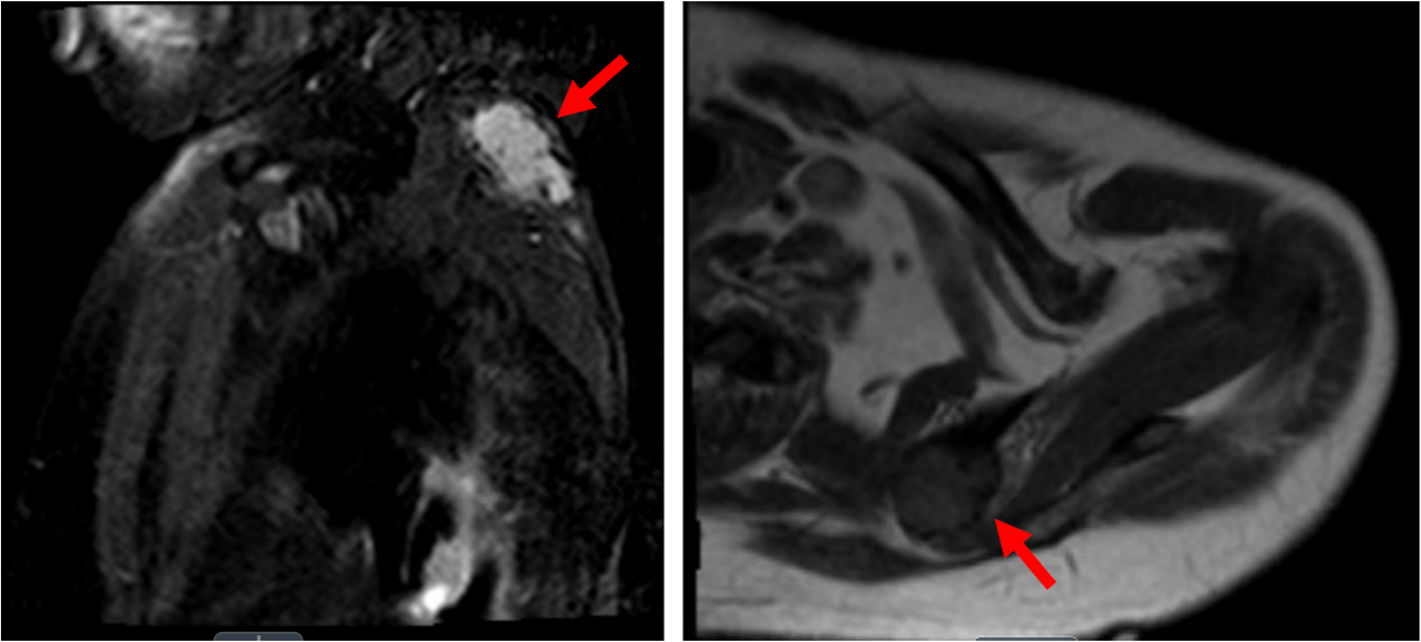
Pre-operative MR scan of patient in case 3 demonstrated a 3.9 × 3.3 × 4.3 cm lobular osteolytic soft tissue mass in the left scapula (red arrows) in both sagittal (left) and transverse planes (right)

A 54-year-old man with a history of diet-controlled type 2 diabetes mellitus and hypertension presented to the University of Michigan Endocrinology clinic for a three-year history of bilateral hip pain due to atraumatic bilateral subtrochanteric stress fractures. He underwent bilateral femoral intramedullary nailing followed by attempted hardware removal of the right cephalomedullary nail. However, the procedure was complicated by a proximal femur fracture and he became wheelchair-dependent. Laboratory evaluation was notable for hypophosphatemia and elevated alkaline phosphatase (Table 1). Unfortunately, he was lost to follow-up for 4 years. He re-established care after developing nontraumatic bilateral rib fractures and a traumatic right periprosthetic femur fracture. Laboratory evaluation at this time noted persistent hypophosphatemia, low 1,25-dihydroxyvitamin D, elevated alkaline phosphatase, and elevated plasma FGF23 levels (Table 1). He was started on phosphorus and calcitriol supplementation. A PET scan demonstrated a focus of increased metabolic activity in the left scapula associated with bony erosion. A CT-guided biopsy of the scapular lesion was consistent with a phosphaturic mesenchymal tumor. Pre-operative MRI noted a 3.9 × 3.3 × 4.3 cm lobular osteolytic soft tissue mass with avid enhancement in the left scapula (Fig. 3). He underwent a left partial scapulectomy. Pathologic examination demonstrated a 4.6 cm mixed connective tissue type phosphaturic mesenchymal tumor with negative surgical margins. Three weeks post-operatively, he reported overall improvement of his body pain and the ability to lift up his arms and legs while sitting in a wheelchair, a significant improvement from his baseline. Upon discontinuation of the phosphorus and calcitriol supplementation, serum phosphorus (4.1 mg/dL) and FGF23 (85 RU/mL) levels normalized. Four months post-operatively, he was able to walk with assistance of a walker.

### Case 4

**Fig. 4:**
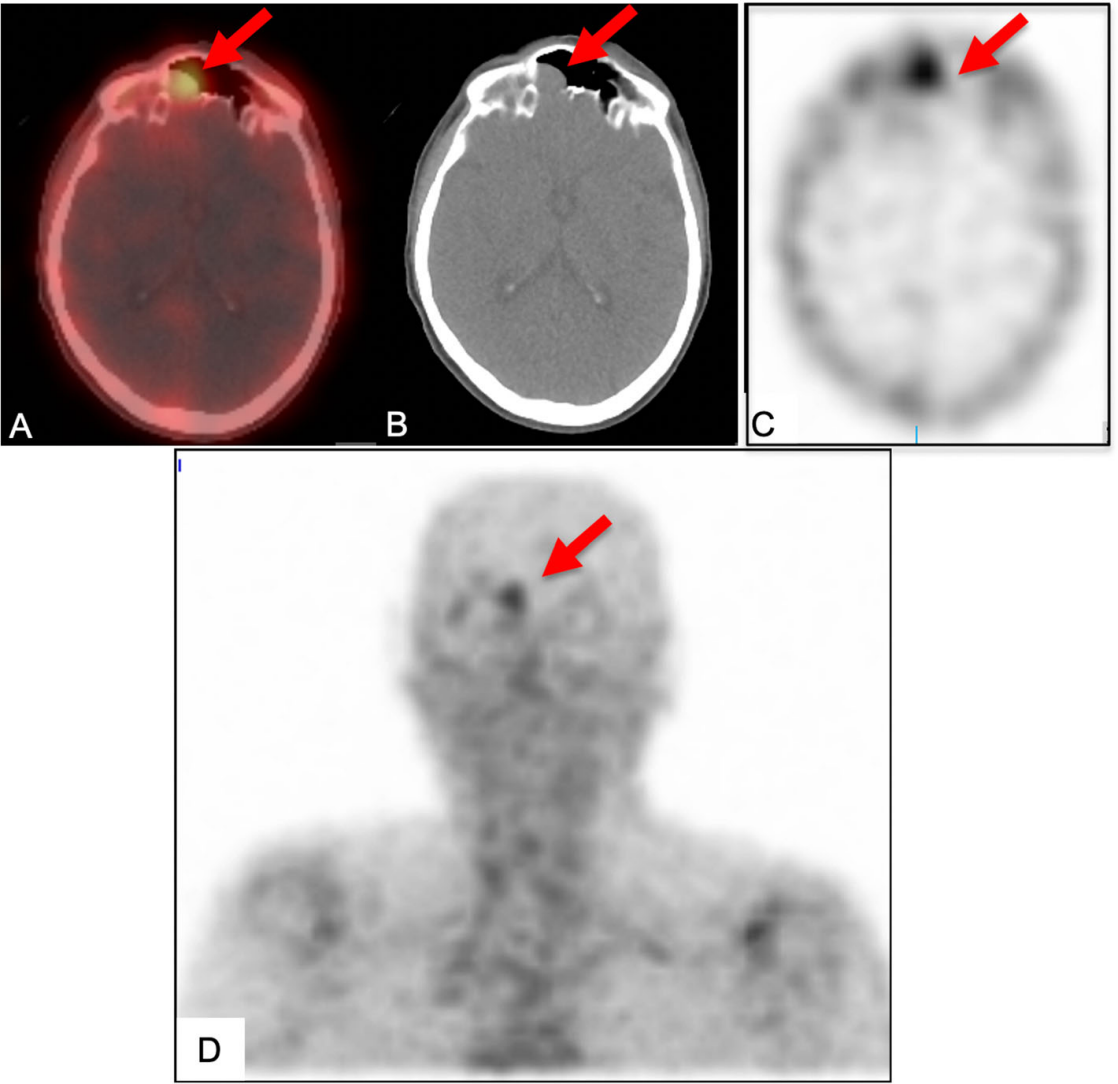
PET (**a**), CT (**b**), and octreotide scans (**c, d**) of patient in case 4, performed at the National Institute of Health, identified a 3 cm right frontal sinus tumor (red arrows) with bone erosion and abutment of the dura

A 61-year-old male presented to the University of Michigan Endocrinology clinic for a three-year history of multiple fractures. These included atraumatic bilateral hip fractures and insufficiency fractures of the lumbar and thoracic vertebrae. Medical history was relevant for colon cancer treated with surgical resection and chemotherapy 11 years prior. One year prior to presentation, he reported diffuse joint pain and ambulatory dysfunction such that he required a walker for ambulation. Bone marrow biopsy was unremarkable with no evidence of malignancy. Laboratory evaluation was notable for hypophosphatemia, low-normal 1,25-dihydroxyvitamin D levels, elevated alkaline phosphatase, and elevated plasma FGF23 (Table 1). Calculated TmP/GFR of 1.43 mg/dL confirmed renal phosphate wasting (Table 2). He was prescribed phosphorous and calcitriol supplementation. Tumor localization studies with ^111^Indium-octreotide and PET scans were initially interpreted as unremarkable with no convincing evidence of a primary tumor. Repeat ^111^Indium-octreotide, PET, and CT scans performed at the National Institute of Health identified a 3 cm right frontal sinus tumor with bone erosion and abutment of the dura (Fig. 4). He underwent a craniotomy with tumor resection and pathologic examination demonstrated a low-grade spindle cell neoplasm consistent with a phosphaturic mesenchymal tumor. Three weeks post-operatively, phosphorus and calcitriol supplementation were discontinued with normalization of the serum phosphorus level (3.8 mg/dL) and decrease in the plasma FGF23 level (405 RU/mL). Four months post-operatively, he was able to ambulate without use of any assistive device and the plasma FGF23 level reached a nadir of 235 RU/mL. A brain MRI 13-months post-operatively revealed only post-operative changes and subtle right frontal dural enhancement.

## Discussion

**Fig. 5:**
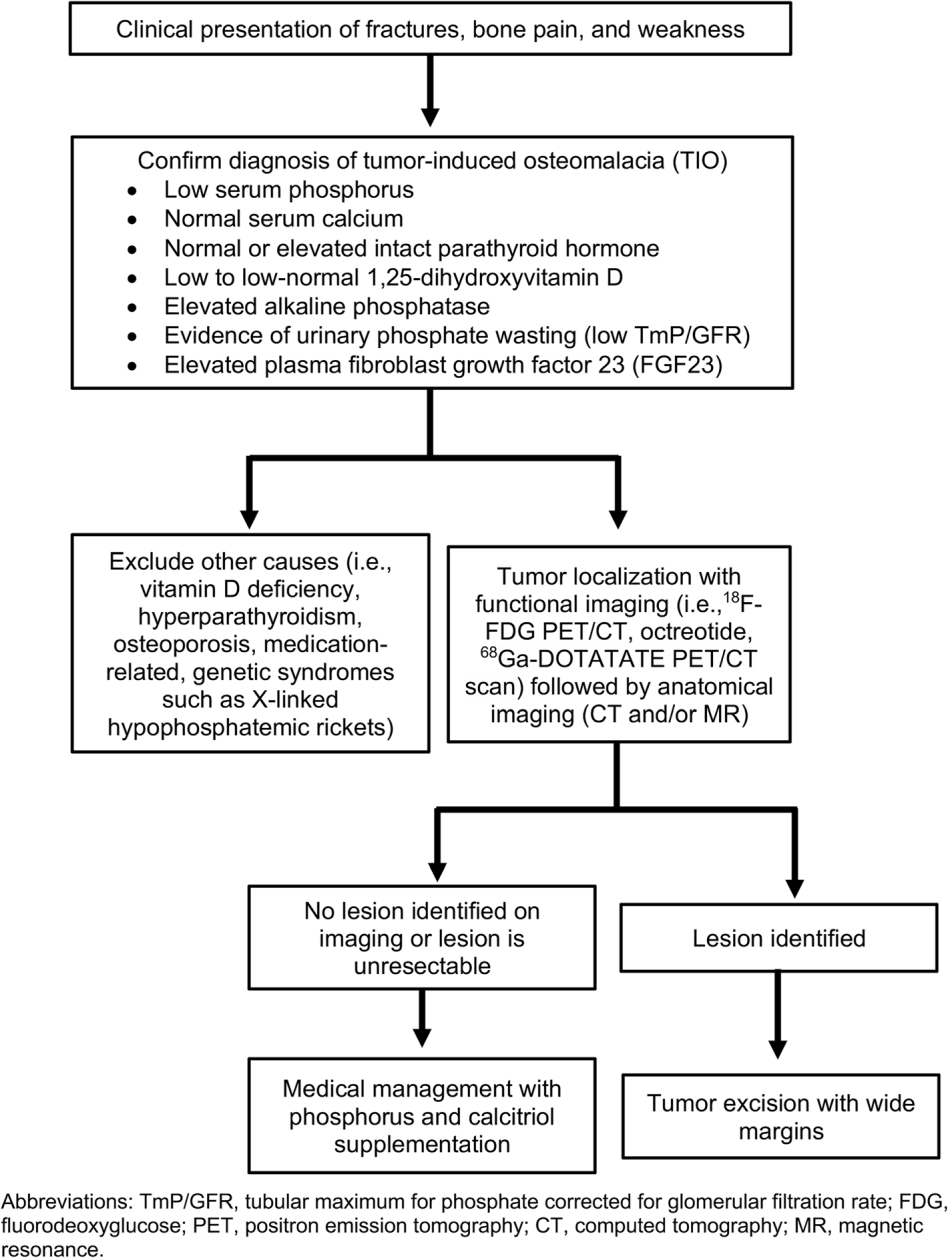
Algorithm for a stepwise approach to the diagnosis and work-up of patients with tumor-induced osteomalacia (TIO)

Tumor-induced osteomalacia is due to tumoral overproduction of the protein FGF23, which acts to inhibit renal phosphate reabsorption in the proximal tubules and suppress 1α-hydroxylase activity, resulting in hypophosphatemia and defective bone mineralization [2, 3, 5, 6]. These four patient cases from a single institution highlight challenges in the diagnosis of TIO that can lead to delayed diagnosis given its rarity, nonspecific symptoms, and omission of serum phosphorus from routine chemistry panels. The essential first step, which is often overlooked in the primary care setting, but should be done in the setting of unexplained fractures, bone pain and weakness, is to check the serum phosphorus level (Fig. 5). Hypophosphatemia associated with fractures should raise suspicion for TIO, and prompt evaluation of renal phosphate wasting with TmP/GFR, which can be derived by nomogram or calculated as (1 – ((urine phosphorus x serum creatinine) / (urine creatinine x serum phosphorus))) x (serum phosphorus) when percent tubular reabsorption of phosphorus is less than or equal to 86% [7, 8]. TmP/GFR is calculated from second morning-void urine and blood samples obtained at the same time, in the fasting state [8]. In TIO, elevated FGF23 causes phosphaturia by reducing expression of the NPT2 sodium-phosphate cotransporter located in the renal proximal tubule, resulting in a low TmP/GFR in the setting of hypophosphatemia. Plasma FGF23 concentrations can be measured using a two-site enzyme-linked immunosorbent assay (ELISA), which is commercially available (Immutopics, Quidel Corporation, San Clemente, CA) [9, 10]. Although confirmation of elevated FGF23 is essential for the diagnosis of TIO, it is important to note that FGF23 is not specific for TIO and can be elevated in other disease processes such as renal insufficiency and X-linked hypophosphatemic rickets (XLH) [11, 12].

The differential diagnosis of hypophosphatemia due to urinary phosphate wasting can be divided into genetic and acquired causes. Inherited conditions include XLH, autosomal dominant hypophosphatemic rickets, autosomal recessive hypophosphatemic rickets, and hereditary hypophosphatemic rickets with hypercalciuria. XLH, which is characterized by an increased plasma concentration of FGF23 due to loss-of-function mutations in the gene encoding phosphate-regulating endopeptidase homolog X-linked (PHEX), is often associated with short stature in children, lower limb deformities, and dental findings on exam such as enamel hypoplasia and dental abscesses or caries [13]. Acquired causes include TIO, renal tubular damage from heavy metal exposure or drugs such as aminoglycoside antibiotics, vitamin D deficiency, and primary or secondary hyperparathyroidism.

As demonstrated in our case series, not all fractures in adults should be assumed to be a manifestation of osteoporosis, which is characterized by low bone mass and skeletal fragility. In TIO, fractures occur as a result of osteomalacia from chronic hypophosphatemia with resultant inadequate mineralization of the bone matrix, which can be worsened when patients are treated with antiresorptive agents such as bisphosphonates. Histologically, osteomalacia is characterized by an increased amount of osteoid, or uncalcified bone matrix, relative to mineralized bone whereas osteoporosis is characterized by a loss of mineralized bone [14]. Pseudofractures, also known as looser zone or Milkman lines, appear as transverse zones of rarefaction on imaging studies and are typically associated with osteomalacia and FGF23-mediated hypophosphatemia. In terms of vertebral fractures, osteomalacic vertebral fractures more often occur in the middle of the vertebra, yielding a fish-mouth deformity, as opposed to the anterior wedging that is more commonly seen in osteoporotic vertebral fractures.

In TIO, tumor localization is essential to achieving curative surgical resection, and a stepwise approach with functional imaging followed by anatomical imaging is recommended (Fig. 5). These tumors, which are typically small in size, have most commonly been identified in the soft tissue and bone of the extremities or appendicular skeleton [15, 16]. There are multiple functional imaging modalities, including ^18^F-FDG PET/CT, octreotide, and ^68^Ga-DOTATATE PET/CT scans. While ^18^F-FDG PET/CT scans can often localize phosphaturic tumors, it is non-specific as it also identifies areas of increased metabolic activity such as actively healing fracture sites [8]. Octreotide scans can be useful for localization because most phosphaturic tumors express somatostatin receptors [17]. There is limited data comparing the different functional imaging modalities. However, recent studies suggest that ^68^Ga-DOTATATE PET/CT scans have higher sensitivity and specificity compared to ^18^F-FDG PET/CT and other somatostatin receptor-based functional scans [17–22]. There is also limited data on the diagnostic utility of selective venous sampling for FGF23. While selective venous sampling may be useful in distinguishing between multiple suspicious tumor sites, it has not been demonstrated to be beneficial in the absence of an identifiable lesion on imaging studies [23].

After the tumor has been localized, complete tumor resection with wide margins is recommended to decrease the likelihood of persistent or recurrent disease (Table 3). In a retrospective review of 230 patients with TIO, Li et al. reported the incidence of persistent and recurrent TIO after the first surgery to be 11.3 and 7.0%, respectively, with the majority of cases due to suboptimal tumor resection [24]. In multivariable analysis, female sex, spine tumors, and bone tissue-involved tumors were identified as factors associated with refractory outcomes [24]. Additionally, each 0.31 mg/dL increase in the preoperative serum phosphorus level (range 0.59 to 2.1 mg/dL) was found to reduce the risk of refractoriness by more than 40% [24]. Post-operative remineralization of the skeleton occurs immediately, but it may take at least one year for significant clinical improvement. Post-operative monitoring of serum phosphorus is necessary to confirm adequate excision of the tumor with the goal of normalization after discontinuation of phosphorus and calcitriol supplementation.

**Table 3:** Current clinical treatment options for tumor-induced osteomalacia (TIO)

Treatment option	When it is appropriate	Recommended Monitoring
Tumor resection with wide surgical margins	In cases of an identifiable lesion on localization studies in patients who are surgical candidates	• Post-operatively, the serum phosphorus is expected to normalize after discontinuation of phosphorus and calcitriol supplementation. • If there is suboptimal tumor resection, monitor for persistent or recurrent TIO
Phosphorus (15–60 mg/kg per day divided into 4–6 doses) and calcitriol supplementation (15–60 ng/kg per day divided into 2–3 doses)	In cases where no lesion is identified on localization studies, complete resection of the tumor is not possible, or the patient is not a surgical candidate	• Monitor serum phosphorus, calcium, intact parathyroid hormone, alkaline phosphatase, and urinary calcium to urinary creatinine ratio • Goal is to maintain serum phosphorus in the lower end of the age-appropriate normal range; serum calcium, parathyroid hormone, and alkaline phosphatase within the normal range; and the spot urine calcium to urine creatinine ratio < 0.2.
Cinacalcet	As adjuvant therapy to phosphorus and calcitriol supplementation	• Monitor urinary calcium for development of hypercalciuria
Burosumab (human monoclonal antibody against FGF23)	This new drug shows promise in treating patients with TIO in whom the lesion cannot be identified or in whom surgical resection is not possible	• In clinical trials, monitoring of serum phosphorus, TmP/GFR, 1,25-dihydroxyvitamin D, and bone turnover markers (procollagen type 1 N-terminal propeptide and collagen type 1 C-telopeptide) is performed

For patients who cannot undergo surgical resection, medical management with phosphorus (dose of 15–60 mg/kg per day divided into 4–6 doses) and calcitriol supplementation (dose of 15–60 ng/kg per day divided into 2–3 doses) is recommended (Table 3) [8, 25]. However, this regimen is often poorly tolerated due to gastrointestinal side effects and can be associated with iatrogenic nephrocalcinosis. Regular monitoring of serum phosphorus, calcium, iPTH, alkaline phosphatase, and urinary calcium to urinary creatinine ratio is necessary. The goal of medical management is to maintain serum phosphorus in the lower end of the age-appropriate normal range; serum calcium, iPTH, and alkaline phosphatase within the normal range; and the spot urine calcium to urine creatinine ratio < 0.2 [2, 8, 25]. In addition to treatment with phosphorus and calcitriol supplementation, adjuvant therapy with Cinacalcet has been demonstrated to increase renal phosphate reabsorption and serum phosphorus levels, with resultant decrease in the dose of phosphorus supplementation required (Table 3) [2, 26].

The FGF23 monoclonal antibody drug, burosumab, was recently approved for the treatment of X-linked hypophosphatemic rickets, and shows promise in treating patients with TIO in whom the tumor cannot be identified or in whom surgical resection is not possible (Table 3) [27–30]. At the 2017 American Society for Bone and Mineral Research (ASBMR) annual meeting, Jan de Beur et al. presented data from an open-label, dose-finding, phase 2 clinical trial of burosumab in 15 patients with TIO. Preliminary data demonstrated that 24 weeks of treatment with burosumab yielded improved mean serum phosphorus, 1,25-dihydroxyvitamin D, and TmP/GFR as well as increased lower limb strength [30].

## Conclusion

Among adults with multiple atraumatic fractures, muscle weakness, and bone pain, the diagnosis of TIO should be considered and serum phosphorus, 1,25-dihydroxyvitamin D, and FGF23 levels checked. Successful tumor localization and excision can lead to cure.

## Data Availability

Data sharing is not applicable to this case report as no datasets were generated or analyzed during the current study.

## Abbreviations

TIO: Tumor-induced osteomalacia
FGF23: Fibroblast growth factor 23
iPTH: Intact parathyroid hormone
TmP/GFR: Tubular maximum for phosphate corrected for glomerular filtration rate
FDG: Fluorodeoxyglucose
PET: Positron emission tomography
CT: Computed tomography
MRI: Magnetic resonance imaging
ELISA: Enzyme-linked immunosorbent assay
PHEX: Phosphate-regulating endopeptidase homolog X-linked (PHEX)
